# Zebularine Boosts Imatinib Efficacy in Cells of Colorectal Cancer via Wnt‐Survivin‐P‐Glycoprotein Pathway

**DOI:** 10.1002/jbt.70885

**Published:** 2026-05-03

**Authors:** Yasmin M. Attia, Abeer Elkhoely, Fatma M. Abdelwahed, Samia A. Shouman, Mervat M. Omran

**Affiliations:** ^1^ Cancer Biology Department, Pharmacology Unit, National Cancer Institute Cairo University Cairo Egypt; ^2^ Pharmacology and Toxicology Department, Faculty of Pharmacy Helwan University Cairo Egypt; ^3^ Cancer Biology Department, Medical Biochemistry and Molecular Biology Unit. National Cancer Institute Cairo University Cairo Egypt; ^4^ Department of Obstetrics and Gynecology University of Chicago Chicago Illinois USA

**Keywords:** apoptosis, colorectal cancer (CRC), imatinib (IM), metastasis, zebularine (ZEB)

## Abstract

Colorectal cancer (CRC) remains one of the leading causes of cancer mortality, with a poor survival rate of less than 15%. Imatinib (IM) and Zebularine (ZEB) alone have shown potential effects in CRC treatment, but their combination has not been thoroughly studied. This study investigates the potential effects of IM and ZEB in colon cancer cells to provide a novel therapeutic agent for managing CRC. Cell growth inhibition, oxidative stress markers, and cell cycle progression were assessed in HCT‐116 cells treated with IM, ZEB, and their combinations. ZEB uptake levels were analyzed by LC‐MS/MS, apoptosis was quantified by flow cytometry, and gene expression changes were analyzed by qPCR. The expression of metastatic markers, apoptotic regulators, and EGFR was assessed. Both IM and ZEB inhibited cell growth in a concentration‐dependent manner, and their combination showed synergistic effects. The combination significantly enhanced oxidative stress. The combination therapy increased apoptosis and necrosis. Furthermore, the combination induced significant S‐phase arrest in the cell cycle. The combination treatment reduced metastatic markers (MMP9, MMP2), and the apoptotic marker Caspase‐9 was upregulated. Additionally, the Bcl‐2 protein, a key regulator of apoptosis, was significantly downexpressed. Remarkably, the combination treatment showed significant inhibition in EGFR levels. Both IM and ZEB combination showed promise in the management of CRC by inducing oxidative stress, promoting apoptosis, and modulating critical genes involved in metastasis and apoptosis. Further investigation will be needed to verify their application in preclinical and clinical settings.

## Graphical abstract

The combination of IM and ZEB showed a promising combination in management of CRC by inducing oxidative stress, promoting apoptosis, and modulating critical genes involved in metastasis and apoptosis.

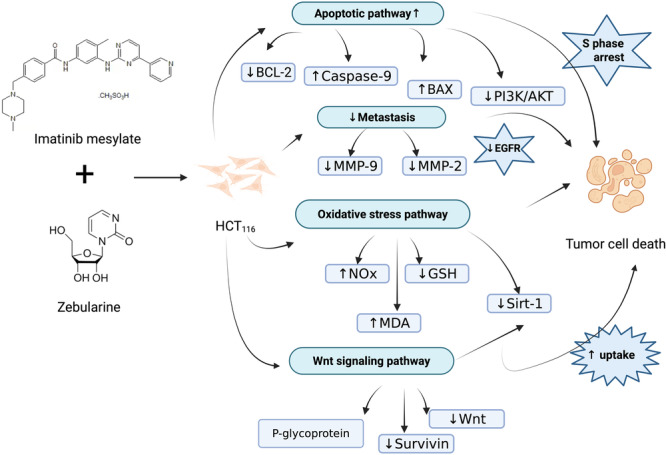

## 1 Introduction

Worldwide, colorectal cancer (CRC) holds the third highest incidence, accounting for 1.9 million new cases reported in 2020, and is the second leading cause of cancer‐related mortality, with nearly 0.94 million deaths [1]. Globally, an estimated 2.5 million new cases are expected in 2035 [2]. Almost more than 25% of advanced CRC cases are diagnosed with metastasis, despite the development of several screening programs to decrease incidence rates of CRC [3, 4]. In addition, difficulties in curative treatment and subsequent tumor‐related mortality have been shown in up to 23% of patients who have undergone curative treatment due to metachronous metastasis development [5]. Chemotherapy represents the cornerstone of CRC treatment with a curative goal in early stages and a palliative role in late stages. Fluoropyrimidine‐based therapies, including capecitabine, have been the main first‐line treatments for CRC for years. However, a combination of 5FU with oxaliplatin and irinotecan‐based therapies resulted in longer median PFS and better OS in metastatic CRC [6]. The use of targeted drugs in clinical practice has been linked to significant improvements in patient survival. Regardless of the advancements in drug discovery for colon cancer, its prognosis remains poor [7]. The repurposed medications strategy explores cost‐effective new uses of traditionally approved medications that could possess a promising role in CRC treatment. Tyrosine Kinase Inhibitors (TKIs) are a cornerstone of precision oncology, playing a crucial role in targeting specific cancer mutations; however, the development of resistance mechanisms has limited the effectiveness of TKIs, which can diminish or negate their therapeutic benefits. Imatinib (IM), which inhibits tyrosine kinases, has been successfully used to treat chronic myeloid leukemia and gastrointestinal stromal tumors since 2006. Previous reports showed good therapeutic efficacy of IM in gastric stromal tumors, leukemia [8], and gynecological tumors [9]. IM is an innovative therapeutic agent designed to target tumor development and cancer progression in individuals at high risk of developing CRC [10]. In mice mimicking human colon cancer, treatment with imatinib (IM) demonstrated the ability to prolong lifespan and enhance survival, particularly in animals with late‐stage tumors and rectal bleeding. It was found that IM significantly reduced tumor proliferation by blocking tumor initiation at the stem cell level [7]. There is a deep need to find new medications for colon cancer, owing to its high death rate, despite the improvements in colon cancer prognosis due to the use of targeted drugs.

Zebularine (ZEB) is a second‐generation, highly stable, potent inhibitor of DNA methylation that preferentially targets cancer cells with minimal side effects. It works by interfering with DNA methylation, a process crucial for regulating gene expression and often altered in cancer [11]. Zebularine can be used alone or in combination with other chemotherapeutic agents. It has the potential to reactivate silenced tumor suppressor genes and enhance the efficacy of other chemotherapeutic agents [12]. It decreases cancer progression and signaling pathways via mechanisms related to proliferation, migration, and invasion. It is often used in combination with other chemotherapeutic drugs due to its ability to potentiate or exhibit synergistic effects [13].

This study tested the efficacy of a combination of IM and ZEB in a colorectal cancer cell line (HCT116), providing a promising basis for clinical investigation and assessing its therapeutic potential in colorectal cancer treatment.

## 2 Materials and Methods

### 2.1 Materials

#### 2.1.1 Culture Conditions of HCT 116 Cells

The human colorectal carcinoma cell line HCT‐116 is a suitable model for studies of tumor behavior and drug efficacy, providing insights into CRC pathogenesis and therapeutic approaches. It was obtained from the American Type Culture Collection (ATCC, Manassas, VA, USA). Cells were sub‐cultivated and maintained in RPMI‐1640 medium (Invitrogen, Carlsbad, CA, USA) in the presence of 1% antibiotic and 10% fetal bovine serum (FBS) from Sigma Aldrich (St. Louis, MO, USA). Cells were incubated in a humidified incubator at 37°C, 5% CO_2_, and 95% air, and maintained at pre‐confluence.

#### 2.1.2 Drugs

##### 2.1.2.1 Imatinib (IM)

IM (C_29_H_31_N_7_O) was obtained from Zhejiang Wuyi Pharmaceutical Co. (Zhejiang, China), and a 1 mM stock solution was prepared by dissolving IM in dimethyl sulfoxide (DMSO) and stored in aliquots at −20°C. Serial dilutions of the compound were prepared in culture media immediately before each experiment (10–200 μM).

##### 2.1.2.2 Zebularine (ZEB)

ZEB was purchased from Sigma‐Aldrich (St. Louis, MO, USA), and a 1‐mM stock solution was prepared by dissolving ZEB in DMSO, then serially diluted in RPMI‐1640 supplemented medium immediately before use to obtain a concentration range of (10–150 μM).

### 2.2 Methods

#### 2.2.1 Determining Cytotoxicity of IM, ZEB, and Their Combinations on HCT116 Cells

The cytotoxicity of IM, ZEB, and their combinations against HCT‐116 was investigated at different drug concentrations using the sulforhodamine B (SRB) assay [14]. Cells were exposed to different concentrations of IM (0–200 µM), ZEB (0–150 µM), and incubated for 48 h.

For the combination regimens, we tested the effect of IC_50_ (8 µM) and IC_25_ (4 µM) of IM with varying concentrations of ZEB (0–150 µM). The surviving fraction was calculated as the ratio of the OD of treated cells to the OD of control cells. The experiments were performed in triplicate, and the results were shown as mean ± SD.

#### 2.2.2 Evaluations of Drug Interaction

Initial dose‐response curves were established for IM and ZEB individually in HCT116 cells. To characterize their interaction, combination indices (CIs) were calculated using CompuSyn software; mechanistic studies employed sub‐IC50 concentrations (4 μM IM and 20 μM ZEB). Combination effects were quantified through fraction affected (Fa) values derived from viability and apoptosis assays, with results presented as Fa‐CI plots and normalized isobologram.

#### 2.2.3 Cell Cycle Analysis

Trypsinization of cells was performed after treatment, and cells were fixed in 70% ethanol (4°C, overnight), washed with PBS, and stained with PI (50 µg/mL) and RNase A (0.1 mg/mL) for 30 min at 37°C. Analysis of samples was performed using an LSR II flow cytometer (BD Biosciences) to quantify G1, S, and G2/M phase distributions.

#### 2.2.4 Determination of Apoptosis

HCT‐116 cells (1 × 10⁶/mL) were grown in 75 cm^2^ flasks for 24 h. Following the manufacturer's guidelines, a fluorescein isothiocyanate (FITC) apoptosis detection kit from BD Biosciences was used to assess cell apoptosis via Annexin V/PI staining. Finally, the FACS Calibur flow cytometer (BD Biosciences) was used to assess apoptotic cells, and the number of apoptotic cells was counted using CellQuest Pro software (BD Biosciences).

#### 2.2.5 Assessment of IM and ZEB Uptake

##### 2.2.5.1 A Liquid Chromatography‐Tandem Mass Spectrometry (LC/MS/MS) Assay Was Used to Analyze the Uptake of IM and ZEB in Cells

To test if the combination of ZEB with IM could affect the intracellular levels of any of them, 2 × 10^4^ cells were cultured per well in 24‐well plates and incubated for 24 h. HCT 116 Cells were incubated with 4 μM of IM, 20 μM of ZEB, and with the combination of both for 48 h. At time 0, 1, 2‐, 4‐, 24‐, and 48 h. Medium was aspirated, centrifuged, and the supernatants were used to determine IM and ZEB concentrations. Extraction and centrifugation at 1400 rpm for 15 min at 4°C were performed on the supernatant as mentioned in Table 1. Injection of the clear supernatant was done into an AB SCIEX LC/MS/MS system (AB SCIEX 3200 QTRAP), considering methods of Determination for both IM and ZEB as illustrated in Table 1. The system is supported by an electrospray ionization (ESI) source and an Agilent 1260 affinity HPLC system. Mass spectrometric analysis was performed in the positive‐ion mode using the Multiquant software program to calculate and analyze results.

**Table 1:** Illustrate the working conditions for the determination of IM and ZEB using HPLC/MS/MS.

	Imatinib	Zebularine
Ion transitions	*m*/*z* 494:394	*m*/*z* 228.8:97.0
Run time	6 min	3 min
Retention time	1.18 min	1.8 min
Injected volume	20 μL	10 μL
Mobile phase	0.1% formic acid in methanol:water (55:45, v/v)	0.1% formic acid in both acetonitril: water (50:50, v/v)
Flow rate	700 μL/min	500 μL/min
Extraction conditions	400 μL of treated media + 1200 μL of methanol	250 μL of treated media + 750 μL Acetonitril
Analytical column	Agilent Poroshell 120‐C18 (50 mm × 3 mm × 2.7 μm, Agilent)	Atlantis T3 (3 μm, 150 × 3 mm)reversed‐phase analytical column (Waters, Ireland)
Calibration range	7.5–240 μmol/L	6.25–100 μmol/L
Reference method	Titier et al. (2005)	Beumer et al. (2006)

1 mg of the drug was dissolved in 1 mL of methanol/water (50:50) to make a stock solution, which was stored in aliquots at −20°C. To prepare working standard solutions of (100 and 10 μg/mL), appropriate dilutions of stock solutions in methanol: water (50:50) were made. Serial dilutions of standards were prepared in drug‐free media and extracted as described in the sample preparation section to produce a chromatogram (Figure S1) and a calibration curve (Figure S2).

#### 2.2.6 Assessment of Total Nitrate/Nitrite (Nox)

HCT116 cells were treated with IM, ZEB, and their combinations for 48 h, then measurements of total nitrate/nitrite (NOx) were performed in cell culture media, according to the method of Miranda [15], using the kit from Cayman Chemical Company (Ann Arbor, Michigan, USA).

#### 2.2.7 Detection of Lipid Peroxidation

To assess lipid peroxidation products in cell lysate, malondialdehyde (MDA) levels were measured in drug‐treated and control cell lysates according to the method of Buege and Aust [16].

#### 2.2.8 Non‐Protein Reduced Thiols Content (Glutathione Content) Determination

To determine reduced glutathione (GSH) content, cell lysates from treated and control cells were used following the Ellman method [17].

#### 2.2.9 Protein Concentration Determination in Media and Cell Lysates

Protein levels in both the medium and cell lysates were measured using the Bradford assay [18].

#### 2.2.10 RNA Isolation and Quantitative Evaluation of Gene Expression Levels of PI3k, AKT Genes, Migratory Genes (MMP‐2, MMP‐9) and Apoptotic Key Regulators (Caspase‐9, Bax, and Bcl‐2)

Total RNA was isolated from HCT‐116 cells treated with IM, ZEB, or their combination, and from untreated HCT‐116 cells, using Direct‐zol RNA Miniprep Plus (Zymo Research Corp., USA, Cat # R2072) according to the manufacturer's instructions. Reverse transcription of total RNA was performed using the miScript RT kit (Qiagen, Hilden, Germany) according to the manufacturer's instructions. Expression levels of mRNA were evaluated using quantitative real‐time PCR using the miScript SYBR Green PCR Kit (Qiagen, Hilden, Germany). The gene expression levels of all genes were normalized to GAPDH. The 2^−^^ΔΔCt^ method was used to calculate gene expression levels [19]. All primers used are listed in Table S3.

#### 2.2.11 Assay of Epidermal Growth Factor Receptor (EGFR)

EGFR levels were assessed in cell lysates using a colorimetric assay kit (Cloud‐Clone Corp., Houston, USA) according to the manufacturer's instructions. EGFR levels were compared to the untreated control level. Triplicate repeats of the experiment were performed.

#### 2.2.12 Survivin, P‐Glycoprotein, Wnt, and SIRT1 Protein Levels Determination

The protein levels of survivin, P‐gp, Wnt, and SIRT1 were evaluated using a Western blotting assay. Cell lysis was performed using RIPA buffer, and the mixture was centrifuged for 15 min at 13,000 rpm. SDS‐PAGE (1% acrylamide) was used to separate the extracted proteins, which were then transferred to a PVDF membrane. Anti‐survivin, anti‐p glycoprotein, and anti‐Wnt (Santa Cruz Biotechnology Inc., CA, USA), anti‐SIRT1 (Cell Signaling Technology Inc, Danvers, MA, USA) primary mouse monoclonal antibodies with 1:500 dilution. Membranes were incubated overnight, then washed and incubated for 1 h at room temperature with an alkaline phosphatase‐conjugated goat anti‐mouse secondary antibody (Novus Biologicals LLC, Littleton, CO, USA) at a dilution of 1:500. The membrane‐bound antibody was detected using a commercially available kit. Win Image Studio Lite 5.2.5 software was used to analyze and measure band intensities using β‐actin (Santa Cruz Biotechnology Inc., CA, USA) at 1:500 dilution as a loading control.

#### 2.2.13 Scratch Wound Healing Assay

Cell migration was evaluated using a scratch wound healing assay, as previously described [20]. HCT116 cells were seeded in 6‐well plates and grown to confluence. A linear scratch was created using a sterile 1‐mL pipette tip, and detached cells were removed by washing twice with warm medium.

Cells were then treated with zebularine, imatinib, or their combination, while 0.1% DMSO served as the vehicle control. Images of the wound area were captured at 0 and 24 h using the same fields and magnification. Cells were fixed with 3.7% paraformaldehyde and stained with 1% crystal violet. Wound closure was quantified using ImageJ software, and the percentage of closure was calculated as:

$\text{Wound closure}(\%)=\frac{A_{0}-A_{t}}{A_{0}}\times 100$

where *A*₀ and *Aₜ* represent wound areas at 0 and 48 h, respectively.

### 2.3 Statistical Analysis

The obtained values were presented as mean ± SD, and differences between groups were analyzed using one‐way analysis of variance (ANOVA) followed by the Tukey–Kramer multiple‐comparison test. A value of 0.05 or less represents a statistically significant difference. Compusyn software was used to determine the interaction between the two drugs in the combination.

## 3 Results

### 3.1 Impact of Different Concentrations of IM or/and ZEB on Cell Viability of the HCT116 Cell Line

**Figure 1:**
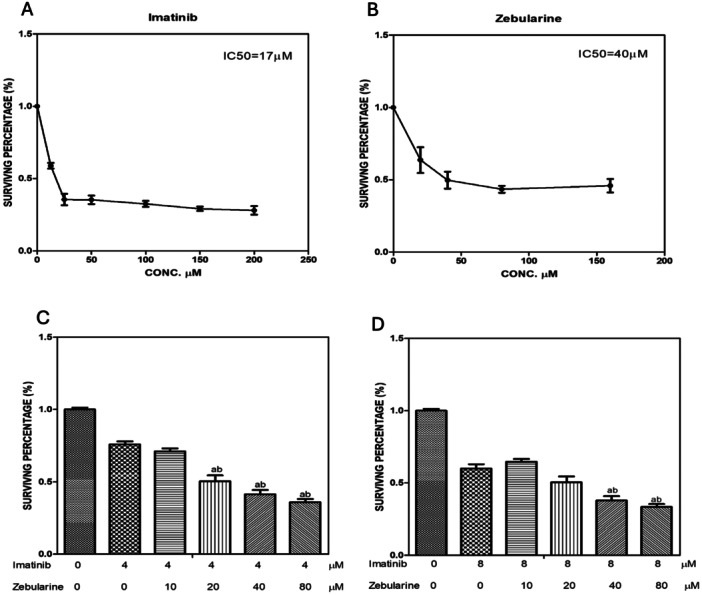
Effect of IC_50_ values of different concentrations of IM (A), ZEB (B), and the effect of 4 µM (C) and 8 µM (D) of IM with different concentrations of ZEB 10‐80 in HCT‐116 cancer cells. The data are presented as means and standard deviations from triplicate observations across three independent experiments.

To test the cytotoxicity of ZEB and IM individually, different concentrations (0–200 µM) of IM and (0–150 µM) of ZEB were applied to HCT‐116 cells. The 50% inhibition (IC50) of IM was 17 µM, while ZEB was 40 µM. Then we aimed to test the optimal lower concentration for each drug that showed better cytotoxicity. Therefore, we used two concentrations of IM (half IC_50_ (8 µM) and quarter IC_50_ (4 µM) with different concentrations of ZEB (0–80 µM) to test the best combination that was used. Interestingly, the IC50 of ZEB decreased from 40 to 20 µM when combined with either the quarter or the half of the IM IC50, as shown in Figure 1.

### 3.2 The IM and ZEB Combination Shows a Synergistic Effect

**Figure 2:**
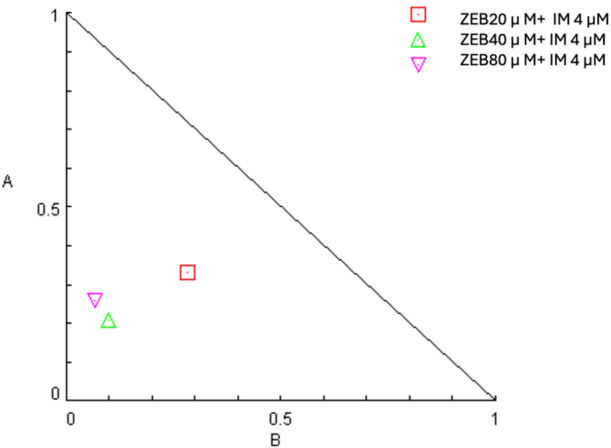
Normalized Isobolgram of the combination of IM and ZEB.

All tested combinations effectively reduced cellular proliferation. Isobologram analysis, conducted using CompuSyn software, was used to determine which drug combinations produced a greater inhibitory effect on cell growth than individual agents alone. The solid line on the isobologram represents the additive effect. Data points located to the left of this line (or below it in a Fa–CI plot) indicate synergism, whereas points to the right (or above in a Fa–CI plot) indicate antagonism (Figure 2). Synergistic interactions, indicated by a combination index (CI) of less than 1, consistently corresponded with a favorable dose reduction index (DRI > 1) for both drugs. Based on these findings, the combination of 4 µM lM and 20 µM ZEB was selected for further mechanistic studies, as it demonstrated a fraction of affected cells (Fa) = 0.5 and a CI = 0.61364, indicating a synergistic effect superior to that of other combinations.

### 3.3 The Combination Regimen Significantly Escalates S‐Phase Arrest

**Figure 3:**
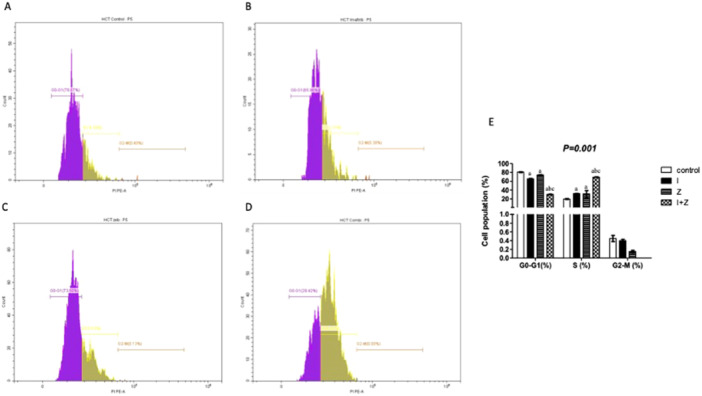
Cell cycle analysis of HCT‐116 cancer cells (A) control, cells treated with (B) IM (17 µM), (C) ZEB (20 µM), (D) their combinations. (E) Bar charts representing the percentage of cell population in G0‐G1, S, and G2‐M phases of the cell cycle of untreated cells and after treatment with IM, ZEB, and their combination. Results are expressed as mean ± SD of two independent experiments performed in duplicate. Statistical significance of results was analyzed using two‐way ANOVA followed by Bonferroni test (a) Significantly different from the control group, (b) Significantly different from IM‐treated group, (c) Significantly different from ZEB‐treated group at *p *< 0.05.

As illustrated in Figure 3, IM caused 32.31% S‐phase arrest. Moreover, treating HCT‐116 cells with ZEB resulted in S‐phase arrest (25.93%), compared with 19.20% in untreated cells. When IM was combined with ZEB, the percentage of cells arrested in S‐phase increased to 69.8%.

### 3.4 The Combination Regimen Significantly Increases Apoptosis

**Figure 4:**
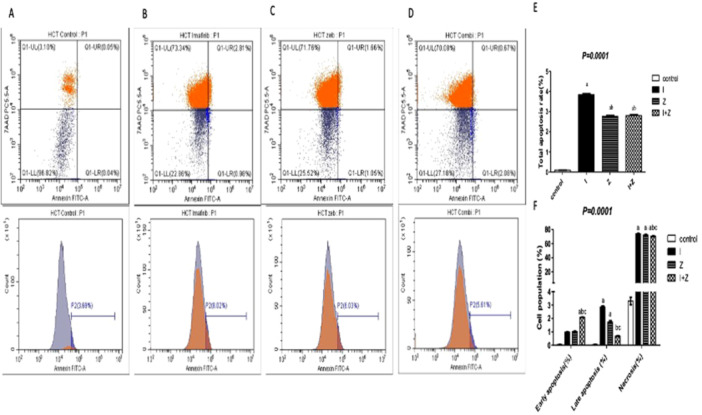
Effect of treatment of IM and ZEB and their combination on total apoptotic ratio using flow cytometry in HCT‐116 cells. Flow cytometry scatterplots for (A) control, (B) IM, (C) ZEB, (D) their combination, (E) quantitative analysis of the total apoptosis rate, and (F) quantitative analysis of early, late apoptosis, and Necrosis rate. Results are expressed as mean ± SD of two independent experiments performed in duplicate. Statistical significance of results was analyzed using one‐way ANOVA followed by Tukey's multiple comparison test and two‐way ANOVA followed by Bonferroni test (a) Significantly different from the control group, (b) significantly different from IM, (c) Significantly different from ZEB treated group at *p *< 0.05.

As shown in Figure 4, the percentages of cells undergoing early apoptosis were 0.99%, 1.025%, and 2% in the IM, ZEB, and combination groups, respectively. On the other hand, the late apoptotic cell percentages were 2.8%, 1.7%, and 0.68% in IM, ZEB, and their combination groups, respectively. Interestingly, the necrotic percentage was highest among the three treatment groups: 73.8%, 72.2%, and 70.54% in the IM, ZEB, and their combination groups, respectively, compared with 3.3% in the untreated group.

### 3.5 Imatinib Modulates HCT‐116 Cellular Uptake of Zebularine

**Figure 5:**
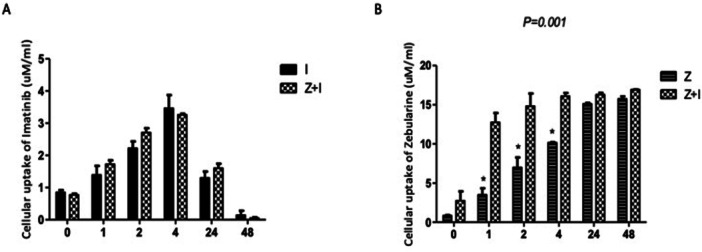
IM (I) regulated cellular uptake of ZEB (Z) in HCT‐116 cells. HCT‐116 cells were treated with 5 μmol/L IM alone, 20 μmol/L ZEB alone, or a combination of both. Medium was then aspirated at 0, 1, 2, 4, 24, and 48 h. IM and ZEB uptake was determined by using liquid chromatography‐tandem mass spectrometry (LC/MS/MS). Results are expressed as mean ± SD of three separate experiments performed in duplicate. Two‐way ANOVA was used to analyze statistical significance, with a repeated and mixed model, followed by the Bonferroni test for adjustment for multiple comparisons, to compare two groups at *p* < 0.05. * Significantly different from the combination.

The effect of IM on ZEB cellular uptake in HCT‐116 cells was examined. It was observed that ZEB uptake increased over time (up to 48 h) in both the ZEB and the combined IM and ZEB groups. A significant increase in HCT‐116 cellular uptake of ZEB was seen in the combination group compared with ZEB alone (*p* = 0.01). While analyzing the impact of ZEB on IM uptake in HCT‐116 cells, no significant difference in imatinib uptake was found. Both groups, the IM and combination, showed a gradual increase in uptake levels over time, reaching a maximum level (3.5 μM/mL at 4 h), then gradually decreasing until 48 h, as shown in Figure 5.

### 3.6 The Combination of IM and ZEB Significantly Raises MDA and NOx and Drops GSH Level

**Figure 6:**
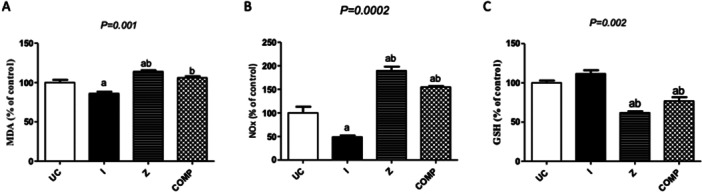
Effect of treatment of IM, ZEB, and their combinations on oxidative stress and antioxidants in the HCT‐116 cell line. MDA (A), NOx (B), GSH level (C). Data were expressed as means ± SD of three independent experiments. a: Significantly different from the control group, b: significantly different from IM at *p* value < 0.05.

The MDA and NOx levels were significantly increased in HCT116 cells treated with ZEB alone (Figure 6A,B), with a remarkable reduction in GSH content compared to normal untreated cells (Figure 6C). On the other hand, treatment with IM significantly decreased MDA and NOx levels (Figure 6A,B), together with a non‐significant increase in GSH content in HCT‐116 cells compared with normal untreated cells (Figure 6C). The IM and ZEB combination showed a 20% increase in MDA and a 105.8% increase in NOx levels compared to IM‐treated cells, and were significantly elevated compared to normal untreated cells (Figure 6A,B). In addition, the combination treatment significantly decreases GSH content by 34.9% compared to IM‐treated cells and the normal control (Figure 6C).

### 3.7 Zebularine Enhances the Effect of Imatinib on Apoptotic and Metastasis Markers

**Figure 7:**
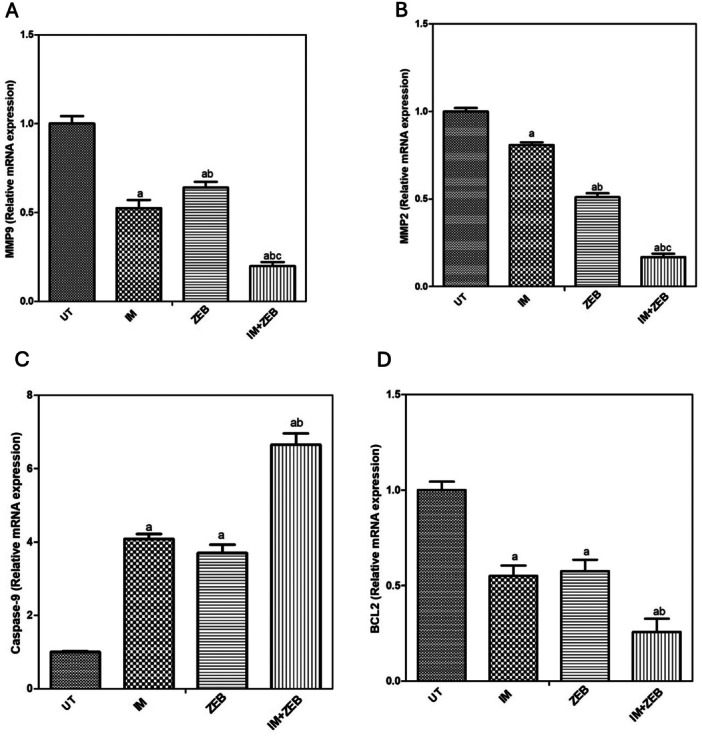
The relative expression of metastatic MMP9 (A), MMP2 (B), and apoptotic Caspase 9 (C), and Bcl2 (D) genes in HCT‐116 cells using qPCR. Cells were treated with 5 μmol/L IM alone or with 20 μmol/L of ZEB alone or a combination of both. Each column represents the mean ± SD of three separate experiments.

The gene expression levels of PI3k, AKT, MMP9, MMP2, Caspase 9, Bax, and Bcl2 were investigated using quantitative real‐time PCR in HCT‐116 cells, as shown in Figure 7. Treating HCT‐116 cells with IM or ZEB resulted in downregulation of the metastatic markers MMP9 and MMP2 (Figure 7A,B). Moreover, the combined therapy enhanced the downregulation effect on both MMP9 and MMP2 genes. Moreover, a considerable upregulation in the expression of apoptotic marker (Caspase‐9) was found in HCT‐116 cells treated with either IM or ZEB alone. At the same time, the combination of IM and ZEB showed the highest upregulation of the Caspase‐9 gene compared to single therapy (Figure 7C). In addition, a remarkable decrease in the antiapoptotic marker (Bcl2) level was observed in HCT‐116 cells treated with IM or ZEB alone. At the same time, it showed the lowest downregulation of the Bcl2 gene expression in the combination therapy compared to single therapy (Figure 7D). Furthermore, there was a slightly upregulation in the proapoptotic Bax gene compared to untreated and imatinib group while zebularine group showed a significant upregulation compared to other groups Figure S4A. As well, combination group showed significant downregulation of PI3K and AKT genes expression levels compared to untreated group Figure S4B,C.

### 3.8 Combination of IM and ZEB Reduced the Level of EGFR in HCT‐116 Cells

**Figure 8:**
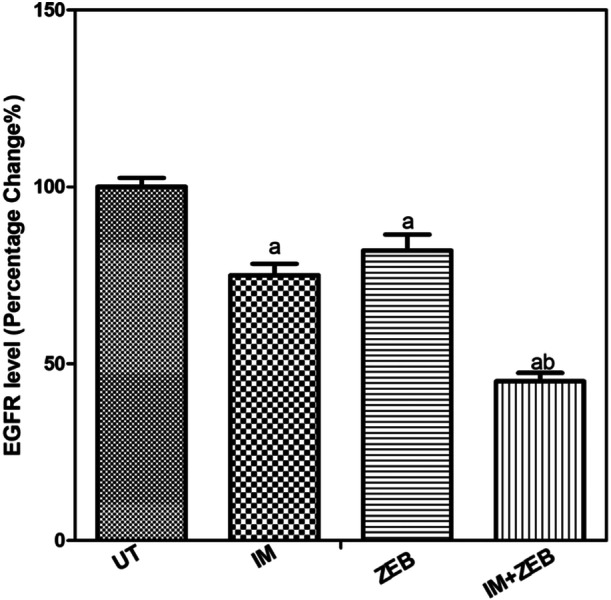
IM combined with ZEB decreases the level of EFGR in HCT‐116 cells. The results were expressed as means ± SD calculated from 3 independent experiments. “a” Significantly different from untreated, “b” Significantly different from HCT‐116 treated with IM, “c” Significantly different from HCT‐116 treated with ZEB at *p* < 0.05.

IM at concentration 4 µM decreased the level of EGFR by 25%, while 20 µM ZEB decreased the level by 18%. Moreover, the combination of both drugs decreased the level by 55% (Figure 8).

### 3.9 The Expression Level of Survivin, P‐Glycoprotein, Wnt, and SIRT1 Proteins in HCT‐116 Cells Treated with Imatinib, Zebularine, and Their Combinations

**Figure 9:**
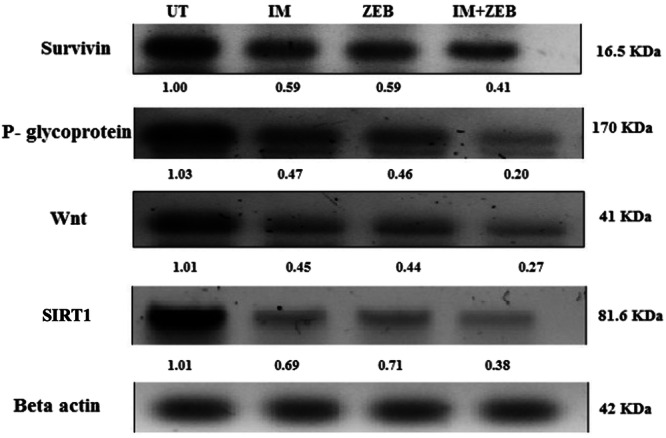
Effect of IM, ZEB, and their combination on the expression level of survivin, P‐glycoprotein, Wnt, and SIRT1 proteins in HCT‐116 cells. Cells were treated with 5 μM IM, 20 μM ZEB, and their combinations. By western blot, the target proteins were detected, normalized to β‐actin, and quantified using ImageJ software. The data were presented as mean ± SD (*n* = 3).

Evaluation of protein expression levels using the western blot assay of HCT‐116 cells treated with IM, ZEB, and their combinations showed downregulation in survivin, P‐glycoprotein, Wnt, Wnt and SIRT1 protein expression, as illustrated in Figure 9. However, reduction levels were more pronounced in the combination group.

### 3.10 Scratch Wound Healing Assay

**Figure 10:**
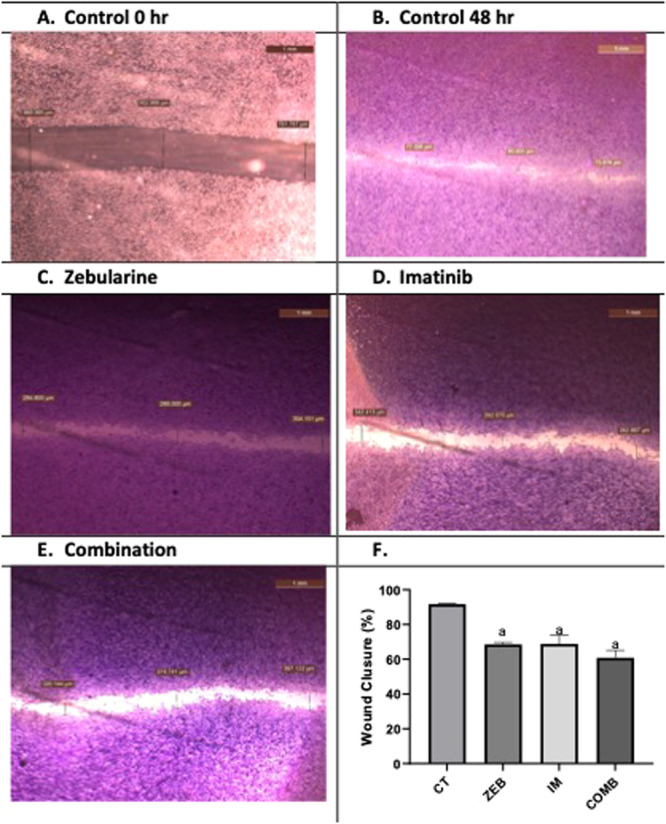
Effects of zebularine and imatinib on HCT116 cell migration evaluated by scratch wound healing assay. Representative phase‐contrast micrographs of scratch wounds in HCT116 cells are shown at 0 h (A) and 48 h (B) under control conditions. Images of cells treated with zebularine (C), imatinib (D), or their combination (E) were captured 48 h after scratch generation. The width of the wound gap was measured using ImageJ software, and quantitative analysis of percentage wound closure is presented in the accompanying bar graph. Data are expressed as mean ± SD from at least three independent experiments. Bars marked with a indicate a statistically significant difference compared with the control group only (*p* < 0.05).

This bar graph shows the percentage of wound (gap) closure measured 48 h after scratching HCT116 cell monolayers under different treatment conditions. Untreated or control cells exhibited the highest wound closure (~90%), indicating strong basal migratory capacity. Then ZEB and IM treatment significantly reduced wound closure to approximately 65%–70%, suggesting inhibition of cell migration. Moreover, the combination of zebularine and imatinib resulted in the lowest wound closure (~60%), demonstrating a greater inhibitory effect on migration than either treatment alone. However, the combination effect did not show a significant reduction from either treatment individually Figure 10.

## 4 Discussion

Despite the latest progress in early diagnosis and proper medications, patients with CRC have a low (<15%) 5‐year survival rate due to the development of resistance to the treatment [21]. This underscores the urgent need for new and improved treatment approaches. In this work, we aim to test the IM and ZEB combination regimen in the colon cancer HCT‐116 cell line, which is characterized by high aggressiveness, limited differentiation, and has an epithelial morphology that metastasizes, making it a suitable model for studying aggressive tumors [22].

Our findings revealed concentration‐dependent inhibition of IM or ZEB, while their combination showed a synergistic effect on HCT‐116 cells at a quarter of IM's IC50 combined with half of ZEB's IC50. Our data demonstrated that IM exerted a concentration‐dependent inhibitory effect on HCT‐116 cell growth. This finding aligns with previous reports highlighting IM's efficacy and potential as an antineoplastic agent in colorectal cancer (CRC) cell lines [23, 24, 25]. It has shown effectiveness in CRC tumors that express c‐KIT or PDGFRA mutations [26, 27]. Moreover, other studies suggested that IM may enhance the effectiveness of conventional chemotherapy regimens or targeted therapies in patients with treatment‐resistant cancer [24, 28]. Moreover, ZEB has shown interest in the field of CRC due to its ability to inhibit DNA methyltransferases and restore the expression of silenced genes as tumor suppressor genes. It revealed a dose‐dependent reduction in cell survival across different cell lines, including colorectal cancer, leading to reduced tumor growth and improved patient outcomes [13]. Early preclinical studies have demonstrated that ZEB can enhance the sensitivity of CRC cells to conventional chemotherapeutic agents and may also have synergistic effects when combined with other targeted therapies [13]. Targeting DNMTs with ZEB reduces colorectal cancer incidence, tumor volume, and mTOR signaling pathway activity [29].

To explore the mechanisms underlying the enhanced cytotoxicity observed with the combination regimen, the intracellular concentrations of both IM and ZEB were determined by LC‐MS/MS. Our data showed a significant increase in ZEB uptake in the combination group compared to cells treated with ZEB alone, indicating that IM may facilitate intracellular accumulation of ZEB or impede its metabolism.

A previous study emphasized the role of human nucleoside transporters (NTs) and organic cation transporters (hOCTs) in modulating the pharmacokinetics and pharmacodynamics of specific DNMT inhibitors (ref). The cellular uptake of ZEB was facilitated by hCNT1, hCNT3, and hENT2. Moreover, hOCT1 and hOCT2 contributed to ZEB efflux, suggesting their function as efflux transporters. Functional alterations due to polymorphic variants of hOCT1 led to a reduction in ZEB efflux [30]. Notably, another investigation demonstrated that the wild‐type hOCT1 efficiently extrudes ZEB, whereas the polymorphic variants C88R, M408V, M420del, and G465R exhibited significantly diminished efflux activity compared to the wild‐type transporter [31]. Although further clinical studies are necessary to confirm this, it can be hypothesized that loss‐of‐function variants may increase drug sensitivity, assuming hOCT1 primarily functions as an efflux rather than an influx transporter.

Conversely, another study indicated that hOCT1‐mediated influx plays a crucial role in the intracellular accumulation of IM and that diminished hOCT1 activity is responsible for reduced in vitro sensitivity to IM [32]. Despite IM being found to be a weak or even non‐substrate for an HOCte in some in vitro studies [33, 34], other reports have shown that the most important contributing factor for IM treatment outcome in patients with CML was hOCT1 expression and its associated activity [35, 36, 37].

Based on this, we propose that hOCTs may function as dual uptake/efflux transporters, potentially contributing to either chemoresistance or chemosensitivity depending on the specific drug or drug combination used in cancer treatment.

Our findings revealed that the combined treatment led to a substantial increase in malondialdehyde (MDA) and nitric oxide (NOx) levels, accompanied by an increase in reduced glutathione (GSH) content compared with IM‐treated cells. This increase in oxidative stress markers in the combination regimen may contribute to the observed cytotoxicity. It was reported that ZEB significantly increased ROS and GSH levels. On the other hand, others reported that ZEB causes depletion of GSH in HeLa cells, an effect that was mitigated by caspase inhibitors [38]. Moreover, treatment with a GSH synthesis inhibitor (L‐buthionine sulfoximine (BSO)) intensified the apoptotic cell death, ROS level, and GSH depletion in ZEB‐treated Calu‐6 cells [39].

Interestingly, recent research has linked IM resistance to elevated GSH levels and heightened activity of glutathione peroxidase and catalase. Given this context, the combination therapy with ZEB holds promise for resensitizing IM‐resistant cells. These findings highlight the potential of this dual approach as a viable strategy for managing challenging cases [40].

Furthermore, our results showed that the combination treatment of IM and ZEB significantly enhanced apoptosis in HCT‐116 cells, as evidenced by increased percentages of cells undergoing early and late apoptosis in the combination group compared to IM or ZEB alone. Interestingly, the necrotic cell percentage was also elevated in the combination group, indicating that the combined treatment induces both apoptotic and necrotic cell death. This dual mode of cell death may contribute to the overall enhanced cytotoxicity observed with the combination regimen [40].

Our data showed the combination of IM and ZEB significantly impacts cell cycle progression, leading to a remarkable increase in S‐phase arrest. Interestingly, it was found that imatinib elevated S phase while reducing G2 phase staining in a dose‐dependent manner, suggesting that IM has anti‐proliferative effects by inhibiting the cell cycle [10]. This compelling observation suggests that the IM‐ZEB combination effectively halts cell cycle progression, thereby inhibiting cell proliferation and contributing to the observed cytotoxic effects. However, a different study reported that ZEB induced cell cycle arrest at the G2/M phase after 72 h of treatment in head and neck cell lines [41], while IM was found to cause G2/M phase arrest in gastric cell lines [42].

At the molecular level, our combination treatment exerts precise control over gene and protein expression, thereby impacting critical pathways involved in apoptosis and metastasis. Our quantitative real‐time PCR analysis reveals specific changes induced by the combination regimen. First, a remarkable reduction in the expression of the metastatic markers MMP9 and MMP2 was observed. These proteins have significant roles in tumor cells’ migration and invasion. Second, the apoptotic marker caspase‐9 is upregulated by the combination treatment. Caspase‐9 is a critical player in apoptosis, and its enhancement supports the observed apoptotic effects. Third, the anti‐apoptotic protein Bcl‐2 is markedly reduced by the combination therapy. This reduction further reinforces the pro‐apoptotic impact. In line with our results, Kim et al. reported that IM induces apoptosis in gastric cancer cells via the JNK/ROS/ER stress pathway [42]. Additionally, ZEB has been shown to increase cell apoptosis and decrease growth through both extrinsic and intrinsic apoptotic pathways in different cell lines [43]. These combined effects likely contribute to the increased apoptosis observed when both drugs are administered together.

Survivin, a multifunctional protein, has an essential role in cancer development. Its overexpression leads to aberrant cell proliferation, apoptosis suppression, and tumor angiogenesis and metastasis [44]. Interestingly, survivin can also exacerbate illness and its impact extends to cancer stem cells, influencing their fate. In light of these findings, therapeutic strategies targeting survivin hold promise for cancer treatment by blocking its expression and inducing cancer cell death [45].

In our recent study, we investigated the combination of IM and ZEB. Notably, SIRT1, another key player, promotes constitutive Wnt signaling—a pathway significantly implicated in tumorigenesis, particularly in breast and colorectal cancer. Moreover, the combination treatment effectively caused downregulation of the expression of survivin, P‐glycoprotein (P‐gp), Wnt, and SIRT1 proteins [46]. P‐gp, a common cause of multidrug resistance, is a member of the ABC transporter family [47]. The observed reduction in these proteins suggests that our combination therapy targets multiple pathways critical for cancer progression. Notably, the synergistic effects of IM and ZEB were more pronounced in the combination group than in the single treatments. These results underscore the potential of IM and ZEB as a promising therapeutic approach, addressing various facets of cancer biology and enhancing treatment efficacy.

Moreover, the combination treatment significantly reduces the level of epidermal growth factor receptor (EGFR) in HCT‐116 cells. IM alone reduced EGFR levels by 25%, while ZEB alone reduced them by 18%. However, the combination treatment resulted in a 55% reduction in EGFR levels. EGFR is a key regulator of cell proliferation and survival, and its downregulation by the combination treatment may contribute to the observed cytotoxic effects.

In conclusion, as summarized in the graphical abstract, our study demonstrates that the combination of IM and ZEB exhibits synergistic cytotoxicity in HCT‐116 cells. The combination treatment enhances cellular uptake of Zebularine, induces oxidative stress, promotes apoptosis, and modulates the expression of key genes and proteins involved in apoptosis and metastasis. These results indicate that the combination of IM and ZEB may represent a promising therapeutic strategy for colorectal cancer. Further studies are warranted to elucidate the underlying mechanisms and to evaluate the efficacy of this combination regimen in vivo.

## Author Contributions

**Fatma M. Abdelwahed:** formal analysis, methodology, writing ‐ original draft, software, writing – review and editing. **Mervat M. Omran:** formal analysis, methodology, writing – original draft, conceptualization, software, data curation, resources, writing – review and editing. **Abeer Elkhoely:** conceptualization, supervision, project administration, visualization. **Yasmin M. Attia:** formal analysis, methodology, writing – original draft, conceptualization, software, data curation, resources, writing – review and editing. **Samia A. Shouman:** supervision, conceptualization, project administration, visualization, writing – review and editing. All authors have read and agreed to the published version of the manuscript.

## Consent

The manuscript was reviewed and approved by all the authors.

## Conflicts of Interest

The authors declare no conflicts of interest.

## Supporting information


**Figure S1:** Chromatograms for detection of (A) Imatinib concentration 35 uM at retention time 0.85 min, (B) Zebularine with concentration 50 uM at retention time 1.8 min.**Figure S2:** Calibration curve for (A) Imatinib at a concentration range (7.5–240 μmol/L) and (B) Zebularine on a concentration range (6.25–100 μmol/L).**Figure S3:** The relative expression of Bax (A), PI3K (B), and AKT (C) genes in HCT–116 cells using qPCR. Cells were treated with 5 μmol/L IM alone or with 20 μmol/L of ZEB alone or a combination of both. Each column represents the mean ± SD of three separate experiments.**Table S1:** Primer sequences.

## Data Availability

The data that support the findings of this study are available on request from the corresponding author.
