# Supplementary material for: Zebularine Boosts Imatinib Efficacy in Cells of Colorectal Cancer via Wnt‐Survivin‐P‐Glycoprotein Pathway
Source: J Biochem Mol Toxicol. 2026 May 3;40:e70885. doi: 10.1002/jbt.70885 (PMC13135721; doi:10.1002/jbt.70885)
Supplement: Supplementary file 1 — Figure S1: Chromatograms for detection of (A) Imatinib concentration 35 uM at retention time 0.85 min, (B) Zebularine with concentration 50 uM at retention time 1.8 min.Figure S2: Calibration curve for (A) Imatinib at a concentration range (7.5–240 μmol/L) and (B) Zebularine on a concentration range (6.25–100 μmol/L).Figure S3: The relative expression of Bax (A), PI3K (B), and AKT (C) genes in HCT–116 cells using qPCR. Cells were treated with 5 μmol/L IM alone or with 20 μmol/L of ZEB alone or a combination of both. Each column represents the mean ± SD of three separate experiments.Table S1: Primer sequences. [file JBT-40-e70885-s001.docx]

**Supplemental Materials**

**
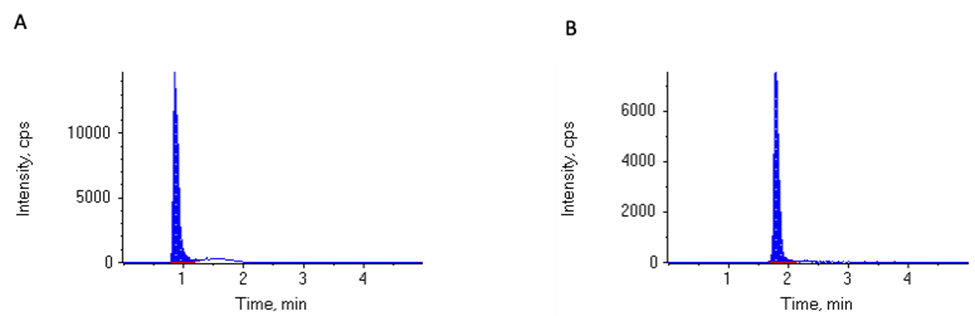
**

**Figure S1:** Chromatograms for detection of (A) Imatinib concentration 35 uM at retention time 0.85 min, (B) Zebularine with concentration 50 uM at retention time 1.8 min

**
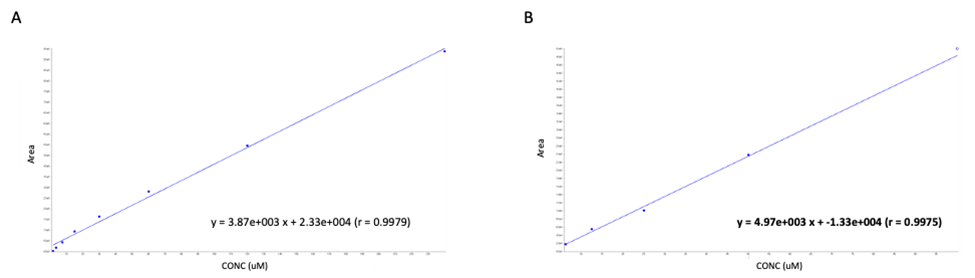
**

**Figure S2:** Calibration curve for (A) Imatinib at concentration range (7.5–240 μmol/L) and (B) Zebularine on concentration range (6.25-100 μmol/L).

**Table S3:** Primer sequences

| **Gene** | **Primer sequence** | **Annealing Temp. (^∘^ C)** |
| --- | --- | --- |
| MMP2 | F: 5’-AGACAGTGGATGATGCCTTTGC-3’  R: 5’-GGAGTCCGTCCTTACCGTCAAA-3’ | 56.6 |
| MMP9 | F: 5’- TTCCAAACCTTTGAGGGCGA -3’  R: 5’- CAAAGGCGTCGTCAATCACC -3’ | 54.8 |
| Caspase 9 | F: 5'‐GTTTGAGGACCTTCGACCAGCT‐3'  R: 5'‐ CAACGTACCAGGAGCCACTCTT‐3' | 57.4 |
| BCL2 | F: 5'‐ CCTGTGGATGACTGAGTACCTG‐3'  R: 5'‐ AGCCAGGAGAAATCAAACAGAGG‐3' | 55.2 |
| GAPDH | F: 5’-ACCCACTCCTCCACCTTTGA-3’  R: 5’-CTGTTGCTGTAGCCAAATTCGT-3’ | 55.1 |
| Bax | F: 5’-AAGCTGAGCGAGTGTCTCAAGCGC -3’  R: 5’-TCCCGCCACAAAGATGGTCACG -3’ | 58 |
| PI3K | F: 5’- GGTTGTCTGTCAATCGGTGACTGT -3’  R: 5’- GAACTGCAGTGCACCTTTCAAGC -3’ | 60 |
| AKT | F: 5'‐ TTCTGCAGCTATGCGCAATGTG ‐3'  R: 5'‐ TGGCCAGCATACCATAGTGAGGT ‐3' | 60 |


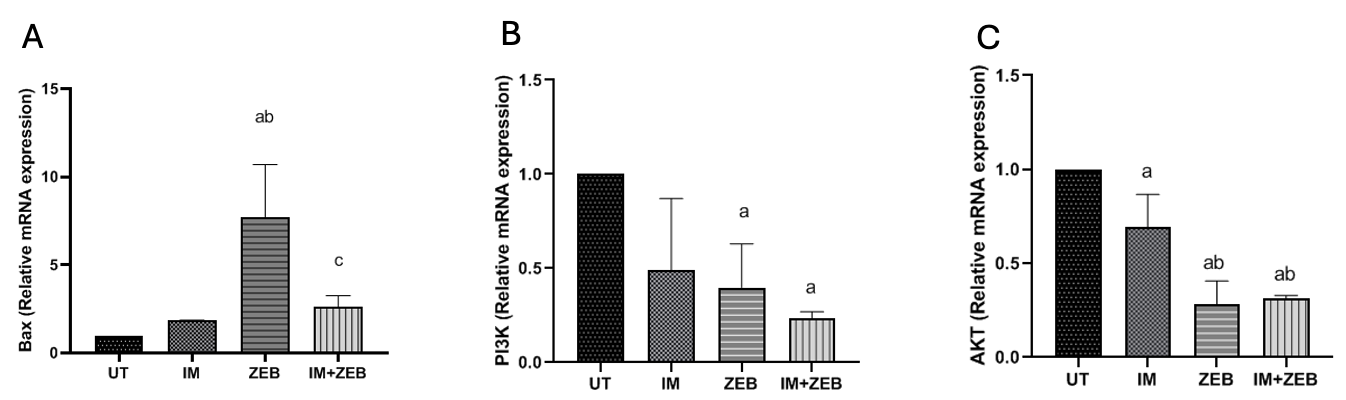


**Figure S4:** The relative expression of Bax (A), PI3K (B), and AKT (C) genes in HCT-116 cells using qPCR. Cells were treated with 5 μmol/L IM alone or with 20 μmol/L of ZEB alone or a combination of both. Each column represents the mean ± SD of three separate experiments.
